# Validation of the Edinburgh Postnatal Depression Scale (EPDS) on the Thai–Myanmar border

**DOI:** 10.1177/0049475517717635

**Published:** 2017-07-12

**Authors:** Harriet Ing, Gracia Fellmeth, Jitrachote White, Alan Stein, Julie A Simpson, Rose McGready

**Affiliations:** 1 Shoklo Malaria Research Unit, Mahidol-Oxford Tropical Medicine Research Unit, Faculty of Tropical Medicine, Mahidol University, Mae Sot, Thailand; 2 Nuffield Department of Population Health, University of Oxford, Oxford, UK; 3 Department of Psychiatry, University of Oxford, Oxford, UK; 4 School of Population and Global Health, University of Melbourne, Melbourne, Australia; 5 Centre for Tropical Medicine and Global Health, Nuffield Department of Clinical Medicine, University of Oxford, Oxford, UK

**Keywords:** Postnatal depression, refugee, migrant, Edinburgh Postnatal Depression Scale (EPDS)

## Abstract

Postnatal depression is common and may have severe consequences for women and their children. Locally validated screening tools are required to identify at-risk women in marginalised populations. The Edinburgh Postnatal Depression Scale (EPDS) is one of the most frequently used tools globally. This cross-sectional study assessed the validity and acceptability of the EPDS in Karen and Burmese among postpartum migrant and refugee women on the Thai–Myanmar border. The EPDS was administered to participants and results compared with a diagnostic interview. Local staff provided feedback on the acceptability of the EPDS through a focus group discussion. Results from 670 women showed high accuracy and reasonable internal consistency of the EPDS. However, acceptability to local staff was low, limiting the utility of the EPDS in this setting despite its good psychometrics. Further work is required to identify a tool that is acceptable and sensitive to cultural manifestations of depression in this vulnerable population.

## Introduction

Mental disorders are among the commonest morbidities of the perinatal period.^1,2^ Consequences of mental disorders such as postnatal depression (PND) are significant for women, their children and society.^3,4^ Infants of mothers with PND experience a higher risk of preterm birth, low birthweight, stunting and poor emotional, cognitive and behavioural development.^4–6^ The prevalence of PND varies globally with estimates ranging between 0.5–60.8%.^7^ Across low- and middle-income countries (LMICs), prevalence has been estimated at 19.8%.^3^ A number of tools exist to screen for PND. One of the most widely used is the Edinburgh Postnatal Depression Scale (EPDS), which has been validated and administered in diverse populations and languages.^8,9^ Its psychometric properties vary across settings with sensitivity and specificity in the range of 59.5–100% and 49–100%, respectively.^9,10^ This variability has been attributed to differences in study methods, participants, cut-off scores and gold standard comparators.^9^

The Thai–Myanmar border is home to an estimated 200,000 migrants and 145,000 refugees from Myanmar, where protracted conflict since 1984 has led to large-scale population displacement into neighbouring Thailand.^11^ In Tak Province, Thailand, where this study took place, displaced communities are predominantly of Karen and Burman ethnicity, each with distinct cultural and language traditions. These displaced populations have previously been shown to experience high levels of depression, anxiety and post-traumatic stress disorder (PTSD) following exposure to forced relocation, violence and long-term socioeconomic disadvantage.^12–14^ High rates of suicidality among women have also been reported in this region, particularly in association with interpersonal violence (IPV).^15^ IPV in turn has been associated with alcohol use, unwanted pregnancy and unsafe abortion, creating a complex interplay between mental health, substance misuse and violence.^16,17^

Our study was based at the Shoklo Malaria Research Unit (SMRU), which has provided maternity care to refugee women in Maela (MLA), the largest refugee camp in Thailand, since 1986 and to rural migrant women at Mawker Tai (MKT) since 1998. MLA and MKT are located 60 km to the north and south, respectively, of Mae Sot, a border town in Tak Province, Thailand. A better understanding of PND in this population is important to identify vulnerable individuals and offer support locally and following repatriation or resettlement. Our study aims to validate Burmese and Karen versions of the EPDS and to assess its local acceptability.

## Methods

We conducted a cross-sectional study of postpartum migrant women attending SMRU clinics at MKT and refugees at MLA. Women were eligible if they were 4–16 weeks postpartum, had given birth to a live, healthy infant, spoke Burmese or Karen, and were willing and able to participate. The EPDS is a ten-item questionnaire which asks women to rate how they have felt during the previous week.^8^ Each item is scored 0–3 with total scores in the range of 0–30 and higher scores representing a higher likelihood of depression. Scores above a cut-off value indicate the need for further assessment. We used the *Structured Clinical Interview for DSM-IV Axis I Disorders* (SCID) as our ‘gold standard’ diagnostic tool for depression.^18^ The SCID evaluates the presence of mental disorders according to established criteria.^19^ For our study, we selected items relating to the diagnosis of major and minor depressive episodes. Translation of the EPDS and SCID into Karen and Burmese was done by SMRU midwives, native speakers of Karen and Burmese and fluent in English and experienced in working with the local community. Back-translations into English were conducted by SMRU staff fluent in Karen, Burmese and English who had not previously seen the original English version. An English-speaking physician compared the original and back-translated English versions to ensure semantic equivalence had been maintained. Owing to the absence of mental health infrastructure in this region, no psychiatrist was available to conduct the translations.

Eligible women were approached by a member of the study team in the MLA and MKT clinic waiting areas and provided with verbal and written information about the study. Women who agreed to participate signed an informed consent form (ICF). Participants with low literacy levels had this read out to them by the study team and those who agreed to participate provided a thumbprint to indicate their consent. A witness signature was also collected for low-literacy participants. Participants were free to withdraw from the study at any time without providing a reason and without effect on their care.

The EPDS was administered in Karen or Burmese in a private room adjacent to the clinic waiting area by SMRU counsellors experienced in working with local pregnant and postpartum women and themselves members of the migrant and refugee communities. All counsellors were fluent in Burmese, Karen and English. Originally, it was planned for high-literacy participants to self-complete the EPDS and for staff to administer the EPDS orally to low-literacy participants. However, it became apparent early in the study that even high-literacy participants found it difficult and distressing to self-complete the EPDS and that their preference was for the questions to be read out to them. Consequently, the EPDS was administered verbally to all participants. Counsellors read the questions out verbatim and provided clarification if required. This method of verbal administration of questionnaires has been used successfully in our setting and amongst other low-income, low-literacy populations.^20–22^

Following completion of the EPDS, women were asked to complete the SCID. The SCID was conducted by an experienced English-speaking physician and four SMRU midwives and counsellors. SCID interviewers were blinded to the results of the EPDS. Women diagnosed during the SCID as having depression were offered counselling and/or medication. Severe cases were given the option of referral to another medical provider. Interviews were carried out between February 2014 and April 2015.

Following completion of the recruitment period, a focus group discussion (FGD) was conducted informally with members of the study team to elicit their views on the administration and appropriateness of the EPDS. Question prompts were used but staff were encouraged to raise issues they felt were relevant or had encountered during the study and to share their opinions honestly.

We used Cronbach’s alpha to determine the reliability of the EPDS in each language. For each cutoff, we calculated sensitivity, specificity, positive predictive value (PPV), negative predictive value (NPV) and the overall proportion of cases correctly identified with corresponding 95% confidence intervals (CIs). We used Youden’s index to identify the optimal cutoff, defined as the value at which (sensitivity + specificity – 1) is maximised.^23^ We assessed criterion validity using receiver operating characteristic (ROC) curves which plot the sensitivity against (1 – specificity). Accuracy of the EPDS (proportion of results correctly identified by the EPDS) was estimated by the area under the ROC curve (AUC). Analyses were conducted using *SPSS 23*.^24^ FGD results were summarised descriptively rather than by formal qualitative analysis owing to the informal nature of the discussion and the small number of participants. Ethical approval was granted by the University of Oxford Tropical Research Ethics Committee (OxTREC 09-14).

## Results

Of 1123 eligible women (121 at MKT; 1002 at MLA), 675 were included, representing 81% (98/121) of eligible women at MKT and 58% (577/1002) at MLA. Baseline characteristics are summarised in Table 1. Of the 670 women for whom complete SCID results were obtained, 14 (six Burmese-speaking, eight Karen-speaking) met the criteria for depression using the SCID, corresponding to a depression prevalence of 2.00% (14/670; 95% CI = 1.0–3.2%) overall, 4.7 (6/127; 95% CI = 1.03–8.4%) in Burmese-speaking participants and 1.48% (8/540; 95% CI = 0.5–2.5%) in Karen-speaking participants. The mean EPDS score was 12.8 (sd 2.7) in women with depression and 2.1 (sd 2.5) in women without depression. A total of 44 participants were aged <18 years, none of whom were diagnosed with depression using the SCID. The mean EPDS score among this group was significantly lower than that of non-depressed women aged ≥18 years (mean [sd] 1.16 [1.55] versus 2.03 [2.38]; *P* < 0.05).

**Table 1.** Baseline characteristics of Karen-speaking and Burmese-speaking participants.

	Karen-speaking (n = 544)	Burmese-speaking (n = 131)	Total (n = 675)
Age (years)*	24 (15–45)	26 (15–41)	24 (15–45)
Gravidity*	2 (1–12)	2 (1–9)	2 (1–12)
Primigravidae	37.9 (206/544)	32.8 (43/131)	36.9 (249/675)
Literate (self-reported)†	61.8 (336/544)	69.5 (91/131)	63.3 (427/675)
Ethnic group^†^			
Karen	96.0 (522/544)	26.7 (35/131)	82.5 (557/675)
Burmese	1.5 (8/544)	32.8 (43/131)	7.6 (51/675)
Burmese Muslim	2.0 (11/544)	32.8 (43/131)	8.0 (54/675)
Other	0.6 (3/544)	7.6 (10/131)	1.9 (13/675)
Smoking^†^	13.4 (73/544)	6.9 (9/131)	12.1 (82/675)

* Results presented as median (range).

† Results presented as % (n/N).

**Figure 1.**
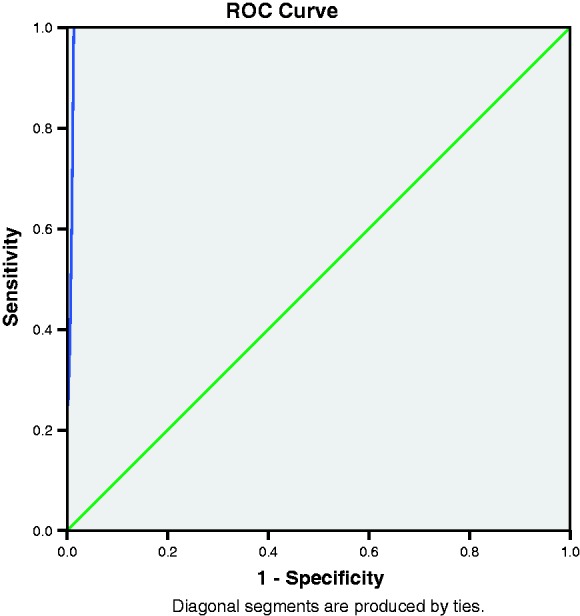
ROC curve for the Karen EPDS.

**Figure 2.**
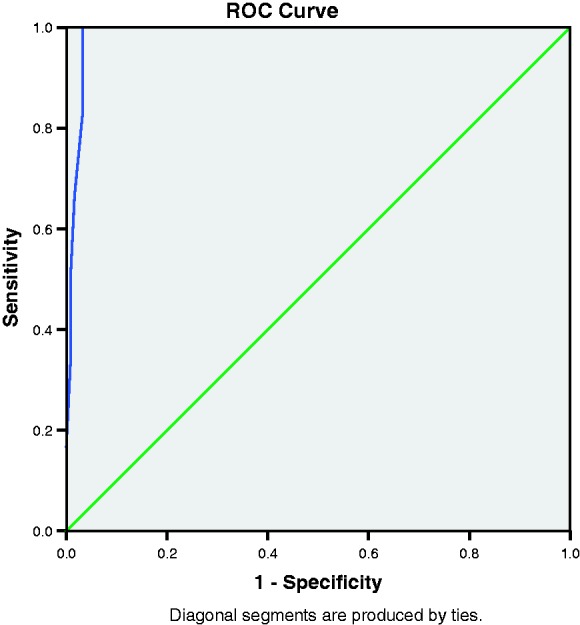
ROC curve for the Burmese EPDS.

SCID and RHS results were complete for 534 Karen-speaking and 128 Burmese-speaking participants. The reliability of the Karen EPDS assessed by Cronbach’s alpha was 0.59. Omitting individual items gave a range of Cronbach’s alpha values of 0.52–0.66. For the Burmese version, Cronbach’s alpha was 0.82 with a range of 0.79–0.82 when individual items were omitted. Tables 2 and 3 show the sensitivity, specificity, PPV, NPV and accuracy by EPDS cutoffs for the Karen and Burmese EPDS, respectively. As expected, sensitivity decreased and specificity increased progressively with increasing cutoffs. PPV was low with a maximum value of 66.7% at EPDS ≥ 13 for the Karen version and 75.0% at EPDS ≥ 14 for the Burmese version. Figures 1 and 2 show the ROC curves for the Karen and Burmese EPDS, respectively. In Karen, the AUC was 0.99 (95% CI = 0.99–1.00) and a cutoff of ≥10 provided 100% sensitivity and 98.7% (95% CI = 97.7–99.7) specificity. In Burmese, the AUC was 0.99 (95% CI = 0.97–1.00) and a cutoff of ≥10 provided 100% sensitivity and 96.7% (95% CI = 93.6–99.9) specificity.

**Table 2.** Sensitivity, specificity, PPV, NPV and accuracy of the Karen EPDS (n = 534).

EPDS cutoff	Sensitivity*	Specificity*	PPV*	NPV*	Accuracy*	Youden’s index
≥ 1	100.0 (100.00–100.00)	39.33 (35.18–43.47)	2.41 (0.76–4.06)	100.00 (100.00–100.00)	40.22 (36.09–44.35)	0.393
≥ 2	100.0 (100.00–100.00)	45.69 (41.47–49.92)	2.68 (0.85–4.52)	100.00 (100.00–100.00)	46.49 (42.30–50.69)	0.457
≥ 3	100.0 (100.00–100.00)	71.35 (67.51–75.18)	4.97 (1.61–8.33)	100.00 (100.00–100.00)	71.77 (67.98–75.56)	0.714
≥ 4	100.0 (100.00–100.00)	78.28 (74.78–81.77)	6.45 (2.13–10.78)	100.00 (100.00–100.00)	78.60 (75.14–82.05)	0.783
≥ 5	100.0 (100.00–100.00)	86.89 (84.03–89.75)	10.26 (3.25–16.99)	100.00 (100.00–100.00)	87.08 (84.26–89.91)	0.869
≥ 6	100.0 (100.00–100.00)	90.07 (87.54–92.61)	13.11 (4.64–21.59)	100.00 (100.00–100.00)	90.22 (87.72–92.72)	0.901
≥ 7	100.0 (100.00–100.00)	94.19 (92.21–96.18)	20.51 (7.84–33.19)	100.00 (100.00–100.00)	94.28 (92.33–96.24)	0.942
≥ 8	100.0 (100.00–100.00)	97.00 (95.56–98.45)	33.33 (14.47–52.19)	100.00 (100.00–100.00)	97.05 (95.62–98/47)	0.970
≥ 9	100.0 (100.00–100.00)	98.13 (96.98–99.28)	44.44 (21.49–67.40)	100.00 (100.00–100.00)	98.15 (97.02–99.29)	0.981
≥ 10	100.0 (100.00–100.00)	98.69 (97.72–99.65)	53.33 (28.09–78.58)	100.00 (100.00–100.00)	98.71 (97.76–99.66)	0.987
≥ 11	62.5 (28.95–96.05)	99.06 (98.25–99.88)	50.00 (19.01–80.99)	99.44 (98.80–100.00)	98.52 (97.51–99.54)	0.616
≥ 12	37.5 (39.50–71.05)	99.44 (98.80–100.00)	50.00 (9.99–90.01)	99.07 (98.25–99.88)	98.52 (97.51–99.54)	0.369
≥ 13	25.0 (0.00–55.01)	99.81 (99.45–100.00)	66.67 (13.32–100.00)	98.89 (98.00–99.77)	98.71 (97.76–99.66)	0.248
≥ 14	25.0 (0.00–55.01)	100.00 (100.00–100.00)	100.00 (100.00–100.00)	98.89 (98.00–99.77)	98.89 (98.01–99.77)	0.250
≥ 15	12.5 (0.00–35.42)	100.00 (100.00–100.00)	100.00 (100.00–100.00)	98.71 (97.75–99.66)	98.71 (97.76–99.66)	0.125
≥ 16	0 (0.00–0.00)	100.00 (100.00–100.00)	100.00 (100.00–100.00)	98.52 (97.51–99.54)	98.52 (97.51–99.54)	0.000

* Values presented as % (95% CI).

**Table 3.** Sensitivity, specificity, PPV, NPV and accuracy of the Burmese EPDS (n = 128).

EPDS cutoff	Sensitivity*	Specificity*	PPV*	NPV*	Accuracy*	Youden’s index
≥ 1	100.00 (100.00–100.00)	42.62 (33.84–51.40)	7.89 (1.83–13.95)	100.00 (100.00–100.00)	45.31 (36.69–53.94)	0.426
≥ 2	100.00 (100.00–100.00)	49.18 (40.31–58.05)	8.82 (2.08–15.56)	100.00 (100.00–100.00)	51.56 (42.90–60.22)	0.492
≥ 3	100.00 (100.00–100.00)	68.85 (60.63–77.07)	13.64 (3.5–23.78)	100.00 (100.00–100.00)	70.31 (62.40–78.23)	0.689
≥ 4	100.00 (100.00–100.00)	73.77 (65.96–81.58)	15.79 (4.2–27.38)	100.00 (100.00–100.00)	75.00 (67.50–82.50)	0.738
≥ 5	100.00 (100.00–100.00)	81.97 (75.15–88.79)	21.43 (6.23–36.63)	100.00 (100.00–100.00)	82.81 (76.28–89.35)	0.820
≥ 6	100.00 (100.00–100.00)	86.89 (80.90–92.88)	27.27 (8.66–45.88)	100.00 (100.00–100.00)	87.50 (81.77–93.23)	0.869
≥ 7	100.00 (100.00–100.00)	90.16 (84.87–95.45)	33.33 (11.55–55.11)	100.00 (100.00–100.00)	90.63 (85.58–95.67)	0.902
≥ 8	100.00 (100.00–100.00)	91.80 (86.93–96.67)	37.50 (13.78–61.22)	100.00 (100.00–100.00)	92.19 (87.54–96.84)	0.918
≥ 9	100.00 (100.00–100.00)	95.08 (91.24–98.92)	50.00 (21.71–78.29)	100.00 (100.00–100.00)	95.31 (91.65–98.97)	0.951
≥ 10	100.00 (100.00–100.00)	96.72 (93.56–99.88)	60.00 (29.64–90.36)	100.00 (100.00–100.00)	96.88 (93.86–99.89)	0.967
≥ 11	100.00 (100.00–100.00)	96.72 (93.56–99.88)	60.00 (29.64–90.36)	100.00 (100.00–100.00)	96.88 (93.86–99.89)	0.967
≥ 12	83.33 (53.51–100)	96.72 (93.56–99.88)	55.36 (23.1–88.02)	99.16 (97.52–100.00)	96.09 (92.74–99.45)	0.801
≥ 13	66.67 (28.95–100)	98.36 (96.11–100)	66.67 (28.95–100.00)	98.36 (96.11–100.00)	96.88 (93.86–99.89)	0.650
≥ 14	50.00 (9.99–90.01)	99.18 (97.58–100)	75.00 (32.57–100.00)	97.58 (94.88–100.00)	96.88 (93.86–99.89)	0.492
≥ 15	50.00 (9.99–90.01)	99.18 (97.58–100)	75.00 (32.57–100.00)	97.58 (94.88–100.00)	96.88 (93.86–99.89)	0.492
≥ 16	33.33 (0.00–71.05)	100.00 (100.00–100.00)	66.67 (13.33–100.00)	96.80 (93.71–99.89)	96.88 (93.86–99.89)	0.333
≥ 17	16.67 (0.00–46.49)	100.00 (100.00–100.00)	100.00 (100.00–100.00)	96.06 (92.68–99.45)	96.09 (92.74–99.45)	0.167
≥ 18	16.67 (0.00–46.49)	100.00 (100.00–100.00)	100.00 (100.00–100.00)	96.06 (92.68–99.45)	96.09 (92.74–99.45)	0.167
≥ 19	16.67 (0.00–46.49)	100.00 (100.00–100.00)	100.00 (100.00–100.00)	96.06 (92.68–99.45)	96.09 (92.74–99.45)	0.167
≥ 20	0 (0.00–0.00)	100.00 (100.00–100.00)	100.00 (100.00–100.00)	95.31 (91.65–98.97)	95.31 (91.65–98.97)	0.000

* Values presented as % (95% CI).

Feedback from study staff obtained through the FGD revealed overwhelmingly negative perceptions of EPDS. Staff described a preference for the SCID questions:

‘*Why can’t we use the other one [SCID] instead? It is easier and better than this one [EPDS].*’

Staff described the questionnaire taking a long time to complete. This was attributed in part to the complexity of explaining the concepts behind some of the items in Karen and Burmese. The meaning of statements such as ‘things have been getting on top of me’, for example, were difficult to convey and had to be explained in detail and using examples in order for participating women to understand the question. Staff also commented on the difficulty in differentiating between EPDS response categories such as ‘quite often’, ‘sometimes’ and ‘not very often’. The general consensus was that differences between these categories were too subtle to describe accurately in Burmese and especially Karen.

‘*It is very hard to find the words to explain the different answers.*’

Even when response categories were adequately conveyed, staff felt that participants often found it difficult to decide which answer applied most to their own frequency of symptoms. They felt it was inappropriate to use a tool that participants found so difficult to complete. All felt that EPDS was not an acceptable tool in this setting. By contrast, feedback on the SCID was positive. Even though there is overlap between the EPDS and SCID in terms of the questions and concepts asked, staff preferred the phrasing of the SCID and the lack of predetermined response categories.

## Discussion

The EPDS is the most widely used tool globally in the assessment of PND. To date, it has never been validated in Karen or Burmese languages or among migrant and refugee women on the Thai–Myanmar border. Our study yields important findings on its psychometric properties and acceptability. The psychometric properties of the translated EPDS, as assessed against translated SCID items on depression, were good; excellent accuracy was found in both languages, and the optimal cut-offs complement findings from other settings.^9,10^ However, while internal consistency was good in Burmese, it was poor in Karen.^25^

There were a number of limitations to our study. First, EPDS and SCID translations as well as the clinical assessments of depression would have been strengthened by input from a local psychiatrist or other mental health specialist. Since no such option was available, we adopted the next best alternative, using a trained physician to conduct interviews with support from local staff who had undergone training from the principal investigator. Second, conducting the SCID with an interpreter poses challenges, with the degree of accuracy and reliability dependent on the skills and experiences of the interpreter. The differences in statistical reliability between Burmese and Karen language versions further highlights the importance of interpretation and communication. For instance, while Burmese has a stronger written tradition, Karen has a stronger oral history and a more limited vocabulary to describe conditions such as depression. The Karen word used most commonly by interpreters to describe depression, tha ba por, means deep sadness and translates literally as a ‘poor heart’. As a result of such language characteristics, communication was indirect in the sense that words or phrases could often not be translated word-for-word but had to be interpreted and given meaning by counsellors. By using our most capable and experienced midwives and counsellors who also had a strong grasp of the English terminology around mental health to work on this study, losses in translation were minimised as far as possible.

The acceptability of an instrument as perceived by local healthcare workers is an important yet often overlooked issue in validation studies.^26^ Studies of acceptability have focused mostly on patients’ perspectives.^27–29^ Yet the perspectives of local staff are vital, especially in low-literacy settings where staff are directly involved in administering questionnaires. The uniformly negative feedback we received from staff was notable, especially in a culture where criticism is rarely overtly voiced. Dissatisfaction with the EPDS in refugee settings has also been reported elsewhere.^30,31^

Our FGD was conducted informally and included only the staff members involved in administering the EPDS. We cannot claim these views to be representative of all staff, and indeed the views of study staff may have been biased given their awareness of the study results. Ideally, we would also have interviewed other (non-study) staff as well as participants. This was not possible owing to staffing and time constraints, but would have enabled a more in-depth understanding of the poor acceptability of the EPDS from both the clinic staff and patient perspectives.

A notable finding of our study is the overall prevalence of PND of 2%. This figure is striking given its place at the low end of global estimates.^7^ It is possible that cases of depression were missed as a result of using the SCID, which has been found elsewhere to identify fewer cases of depression than expected.^32^ Somatic symptoms of mental disorders, which are more common in non-Western cultures, may also have been missed.^3,22,33^ In a qualitative study exploring women’s perceptions of mental illness on the Thai–Myanmar border, common symptoms of depression described included a ‘heavy head’, tingling and numbness.^34^ These symptoms may not have been identified as ‘depressed’ on the SCID.

The finding that rates of depression and EPDS scores were significantly lower among mothers aged <18 years is of interest given the risks associated with pregnancy at a young age. Younger women may have felt less confident in reporting symptoms of depression. An in-depth discussion with this group may have helped to clarify whether a different set-up, for example the presence of a younger member of the counselling team, may have put these women more at ease.

## Conclusion

PND is a significant problem worldwide with potentially long-lasting consequences for the mother, her children and wider society. There is an urgent need for a better understanding of the validity and acceptability of screening tools across different cultures and languages in order to detect women with mental distress and provide appropriate support prior to and following resettlement. Given the associations between perinatal depression and interpersonal violence documented in many low-resource and conflict-affected settings including the Thai–Myanmar border area, identifying women with depression at an early stage is an essential component in ensuring not only their general wellbeing but also their safety.^15,35–37^ Our study highlights the significant challenges of diagnosing conditions such as depression in low-resource, low-literacy settings. Although the EPDS has been widely described as being easy to administer, our results do not support this. In our setting, the EPDS was psychometrically valid but its low acceptability to local staff makes it an inappropriate tool. Further work is required to develop a tool that is acceptable and sensitive to culturally specific manifestations of PND, especially in this population.
